# Willingness to use a wearable device capable of detecting and reversing overdose among people who use opioids in Philadelphia

**DOI:** 10.1186/s12954-021-00522-3

**Published:** 2021-07-23

**Authors:** Katie Kanter, Ryan Gallagher, Feyisope Eweje, Alexander Lee, David Gordon, Stephen Landy, Julia Gasior, Haideliza Soto-Calderon, Peter F. Cronholm, Ben Cocchiaro, James Weimer, Alexis Roth, Stephen Lankenau, Jacob Brenner

**Affiliations:** 1 Perelman School of Medicine, University of Pennsylvania, 3450 Hamilton Walk, Stemmler Building, Office #220, Philadelphia, PA 19104 USA; 2 Ballinger, 833 Chestnut Street, Suite 1400, Philadelphia, PA 19107 USA; 3 Penn Department of Medicine Clinical Trials Unit, Anatomy-Chemistry Building, 1st Floor, Philadelphia, PA 19104 USA; 4 Department of Family Medicine and Community Health, Perelman School of Medicine, University of Pennsylvania, Philadelphia, PA USA; 5 Center for Public Health Initiatives, Perelman School of Medicine, University of Pennsylvania, Philadelphia, PA USA; 6 Leonard Davis Institute of Health Economics, Perelman School of Medicine, University of Pennsylvania, Philadelphia, PA USA; 7 Prevention Point Philadelphia, 2913-15 Kensington Ave, Philadelphia, PA 19134 USA; 8 Penn State College of Medicine, 500 University Dr, Hershey, PA 17033 USA; 9 Department of Computer and Information Science, University of Pennsylvania, Levine Hall, 3330 Walnut St, Philadelphia, PA USA; 10 Dornsife School of Public Health, Drexel University, Nesbitt Hall, 3215 Market St, Philadelphia, PA 19104 USA; 11 Perelman School of Medicine, University of Pennsylvania, 3400 Civic Center Blvd, Philadelphia, PA 19104 USA

**Keywords:** Naloxone, Overdose detection, Overdose reversal, Opioid use disorder, Medical device, Wearable device, Substance use disorder

## Abstract

**Background:**

The incidence of opioid-related overdose deaths has been rising for 30 years and has been further exacerbated amidst the COVID-19 pandemic. Naloxone can reverse opioid overdose, lower death rates, and enable a transition to medication for opioid use disorder. Though current formulations for community use of naloxone have been shown to be safe and effective public health interventions, they rely on bystander presence. We sought to understand the preferences and minimum necessary conditions for wearing a device capable of sensing and reversing opioid overdose among people who regularly use opioids.

**Methods:**

We conducted a combined cross-sectional survey and semi-structured interview at a respite center, shelter, and syringe exchange drop-in program in Philadelphia, Pennsylvania, USA, during the COVID-19 pandemic in August and September 2020. The primary aim was to explore the proportion of participants who would use a wearable device to detect and reverse overdose. Preferences regarding designs and functionalities were collected via a questionnaire with items having Likert-based response options and a semi-structured interview intended to elicit feedback on prototype designs. Independent variables included demographics, opioid use habits, and previous experience with overdose.

**Results:**

A total of 97 adults with an opioid use history of at least 3 months were interviewed. A majority of survey participants (76%) reported a willingness to use a device capable of detecting an overdose and automatically administering a reversal agent upon initial survey. When reflecting on the prototype, most respondents (75.5%) reported that they would wear the device always or most of the time. Respondents indicated discreetness and comfort as important factors that increased their chance of uptake. Respondents suggested that people experiencing homelessness and those with low tolerance for opioids would be in greatest need of the device.

**Conclusions:**

The majority of people sampled with a history of opioid use in an urban setting were interested in having access to a device capable of detecting and reversing an opioid overdose. Participants emphasized privacy and comfort as the most important factors influencing their willingness to use such a device.

***Trial registration*:**

NCT04530591.

**Supplementary Information:**

The online version contains supplementary material available at 10.1186/s12954-021-00522-3.

## Background

The USA has recorded unprecedented increases in drug overdose mortality over the past 30 years, especially related to the class of opioids that includes prescription analgesics (e.g., oxycodone), heroin, and fentanyl [1]. According to the Centers for Disease Control and Prevention (CDC), 47,600 people died of an opioid overdose in the USA in 2017 alone, representing over two-thirds of all drug overdose deaths [2]. Rates of fatal overdose due to synthetic opioids have risen 10% between 2017 and 2018 in the USA [2], owing to increased contamination of opioid supplies with fentanyl, a drug that is 30 to 50 times more powerful than heroin [3]. The CDC reports that the number of overdose deaths attributable to fentanyl increased by over 1100% between 2013, when the increase began, and 2016 [4]. The rising rates of overdose have become particularly pronounced over the course of the COVID-19 pandemic, as rates of relapsing opioid use disorder (OUD) soar [5]. Non-fatal overdoses also cause significant physiological complications, including brain hypoxia, and have been correlated with decreased cognitive performance, increased depressive symptoms, and increased suicidal ideation [6]. Those who experience a non-fatal overdose are at greater risk of experiencing a subsequent overdose, both fatal and non-fatal. Harm reduction efforts to mitigate the long-term effects of opioid overdose are essential, with estimates of overdose rates—including both fatal and non-fatal—among people who inject drugs as high as one in five each year [7].

Opioid overdoses cause morbidity and mortality by depressing an individual’s respiratory drive, leading to hypoxemia and eventually death. The current standard of care for severe opioid overdose is administering naloxone, an opioid-receptor antagonist that counteracts opioid-induced respiratory depression [8]. Naloxone is FDA-approved for intranasal, intramuscular, intravenous, and subcutaneous delivery and can reliably reverse opioid overdoses within minutes. In states most affected by the opioid epidemic, initiatives to reduce opioid overdose mortality have focused on promoting judicious opioid prescribing practices and the widespread distribution of lay and first-responder naloxone kits. Prior work suggests programs for community-delivered naloxone are safe public health measures that have the potential to reduce rates of fatal overdoses [9].

However, one major limitation of existing naloxone distribution efforts is that the successful reversal of an opioid overdose currently requires identification and reversal by another person. One study found that 51.8% of fatal overdoses were not witnessed by another individual, while 27.4% of fatal overdoses were witnessed by a bystander who failed to recognize the symptoms of opioid overdose [10]. Even when an overdose is detected, first responders may not arrive until it is too late, especially in rural areas [11]. Surprisingly, there is no current means to address opioid overdoses in cases where people use alone [12, 13]. Previous efforts to target such populations have relied on mobile applications, which, while potentially beneficial, still require bystander intervention and high community trust [14–17]. Reliable methods of detecting severe opioid overdoses in community settings could address these challenges. In pursuit of advanced medical devices and analytics, the FDA launched a Innovation Challenge in May 2018 to incentivize development of technologies to address the addiction crisis [18]. Alternative device-based strategies to eliminate the need for bystander intervention are being actively studied, including the use of biosensors to detect physiological changes after opioid use [19–23] and autoinjectors to reverse overdose [24], but user acceptability of such solutions remains an open question.

While stand-alone studies of willingness to use a biosensor or mobile application-based bystander network have been pursued, there is a lack of data to understand user perception and need for naloxone options that do not require bystander intervention [15, 25]. In this study, we investigated the preferences of individuals who use opioids regarding a wearable device capable of detecting and reversing overdose without bystander intervention. We hypothesized that demand for such a device would be strong, especially among those with previous overdose experiences. By enhancing our understanding of user preferences for a wearable overdose detection and reversal device, we hope to highlight the need to address the risks to people who use alone and amplify the impact of community-based naloxone programming.

## Methods

We used a cross-sectional survey (*n* = 97) in combination with a semi-structured interview (*n* = 95) to evaluate the preferred methods for automated opioid overdose detection and reversal agent delivery among people who use opioids in the Kensington neighborhood of Philadelphia, PA, USA. Philadelphia is home to a particularly vulnerable population, where 75–80% of fatal opioid overdoses occur alone in the home and opioid-related EMS responses since the March 2020 COVID-19-related stay-at-home mandates have been rapidly increasing [26]. Kensington is recognized as one of the epicenters of the opioid crisis nationally, with over 1000 overdose deaths in both 2017 and 2018 [27].

### Survey

A 50-item questionnaire was administered to assess the acceptability of various overdose detection methods in isolation (“sensing-only” devices) and various functional devices in comparison with sensing-only devices. Questions regarding opioid use history, including frequency of use, time of use, and effect of use on behaviors and socialization, were asked in a manner paralleling the structure of the 1984 American Drug and Alcohol Survey [28]. Participants were also asked questions regarding past experience with opioid overdose; those participants who reported having experienced an overdose were asked additional questions about frequency of overdose, bystander intervention, and need for post-overdose medical care. A five-point Likert scale was used to quantify preferences for various sensing-only devices (nasal cannula, wrist bracelet, skin patch, ankle strap, etc.) and other functional devices, such as a device that alerts bystanders to an overdose event, a device that straps naloxone to the body for bystander administration, and a device that administers naloxone upon overdose detection, among others. In addition, demographic data including age, race and ethnicity, sex, employment status, and household income were collected from all participants. The questionnaire was administered verbally by a study coordinator or in written form, depending on participant preference. The survey is fully detailed in “Appendix 1.”

### Semi-structured interviews

To specifically understand the acceptability of a device that senses opioid overdose and injects naloxone in response, we conducted semi-structured interviews with study participants. The deltoid and quadriceps muscles are commonly recognized as two potential sites for intramuscular emergency naloxone injection [29]; as such, the interviews were focused on user preferences and usage behaviors for a device that would be strapped superficially to the thigh or shoulder. Participants were asked questions regarding comfortability, size preference, propensity for misplacement, and the importance of various aspects of the device’s appearance. We supplemented the interview with non-functional prototypes of varying size to give the participants a tactile experience of how the device may eventually function. The questions from the semi-structured interview are detailed in “Appendix 2,” and the prototypes are illustrated in Additional file 1: Figure 1.

### Study participants

Participants were enrolled in August and September 2020 at three Prevention Point Philadelphia (PPP; Philadelphia, Pennsylvania, USA) program sites—a respite center, a homeless shelter, and a harm reduction drop-in center. At the drop-in center, participants were recruited at the time of syringe exchange events. Inclusion criteria included age of 21 years or older, ability to provide informed consent, and one of the following: (1) self-reported opioid use history of 3 months or longer, (2) a recent or upcoming surgery where opioids were or will be administered, or (3) moderate to severe chronic pain treated with prescription or illicit use of opioids. Those who self-reported pregnancy upon screening for study participation were excluded due to IRB concerns. Participants were compensated $10 for completion of the survey only and $15 for completion of the survey and the semi-structured interview via preloaded debit cards. The survey was administered after enrollment and the semi-structured interview followed immediately afterward. All participants were offered participation in both the survey and semi-structured interview portions of the study.

### Analysis

The primary outcome of interest was the proportion of respondents indicating likelihood to wear devices with different proposed functionalities and sensors at different proposed locations. To capture and present likelihood that proposed devices would be worn, quantitative responses on Likert scales from 1 (very unlikely) to 5 (very likely) were grouped into the binary categories “likely” (4 or 5) and “unlikely” (1, 2, or 3). We considered the likelihood of using each proposed device and compared these for both device functionality and sensing location. Individual-level factors associated with likelihood of using a device that senses and reverses opioid overdose were assessed using Chi-squared tests or logistic regression as appropriate.

The primary focus of the semi-structured interview was feedback to inform design constraints of a non-functional prototype device with the specific proposed functionality of accurate overdose detection and reversal. Quantitative survey questions on Likert scales were combined with open-ended feedback questions. All quantitative responses were grouped into binary categories and interpreted qualitatively. Elicited free-response feedback was grouped thematically, reported, and discussed as deemed pertinent to design constraints. Data were integrated qualitatively, as both Likert scale categorical data and open-ended responses were considered within the identified themes.

## Results

### Study participants

**Fig. 1:**
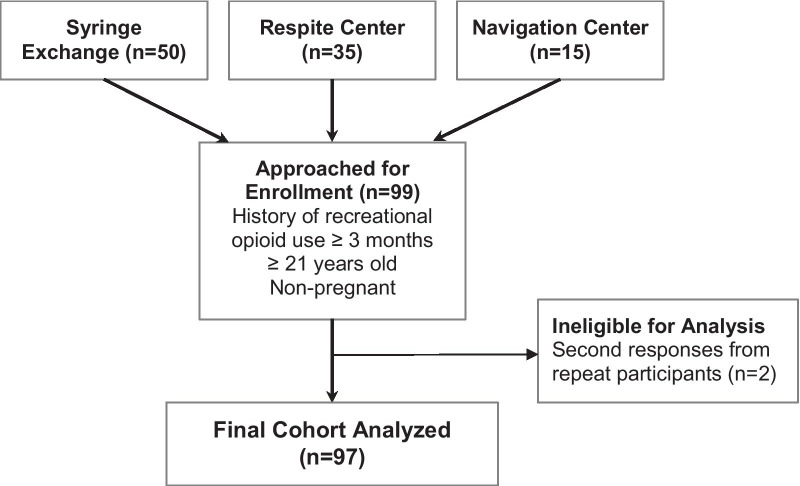
Consort diagram of inclusion and exclusion criteria

Patient inclusion and exclusion are demonstrated in Fig. 1. Ninety-nine patients enrolled in the analysis and completed both the survey and interview components of the study. Two participants were sampled twice, and their second surveys and interviews were discarded. Participant demographic information is described in Table 1. The study sample was mostly white (47%) and majority male (59%). Participants reported a median duration of opioid use of 12 years (IQR: 8–20 years), with 71% of participants reporting one or more lifetime overdoses. Among those who had previously overdosed, the median estimated number of overdoses was 3 (IQR: 2–7). A slim majority of participants (55%) reported a self-perceived high likelihood (4 or 5 on a five-point Likert scale) of being in a location where a bystander would be likely to administer naloxone in the event of an overdose. Participants were not asked clarifying questions regarding who these bystanders would be, such as if they used opioids with others or in a public location.

**Table 1:** Demographic characteristics and opioid use history of study participants

Demographic table	
Age [*median *(*IQR*)]	41 (37–49)
Male	57 (58.8%)
**Race**	
White/non-Hispanic	46 (47.4%)
Black/non-Hispanic	24 (24.7%)
Black/Hispanic	6 (6.2%)
Other/Hispanic	17 (17.5%)
Other/non-Hispanic	3 (3.1%)
Native American	1 (1.0%)
**Housing status**	
Homeless	31 (32.0%)
House	7 (7.2%)
Apartment	8 (8.2%)
Shelter	36 (37.1%)
Unknown	15 (15.5%)
**Opioid use history**	
Started after injury/surgery	46 (47.4%)
Average length of use (years) *[median (IQR)]*	12 (8–20)
**# of days/week of use**	
Unknown	1 (1.0%)
1	0 (0.0%)
2–3	9 (9.3%)
4–6	7 (7.2%)
7	80 (82.5%)
**# of times/day of use**	
Unknown	2 (2.1%)
1	1 (1.0%)
2–3	21 (21.6%)
4–6	46 (47.4%)
7+	27 (27.8%)
Been to an inpatient rehab	79 (81.4%)
Used MOUD	84 (86.6%)
High likelihood of bystander intervention with naloxone (4/5)	53 (54.6%)
**Overdose history**	
Friends/family have overdosed	77 (79.4%)
Friends/family have overdose complications	56 (57.7%)
Friends/family have died from overdose	75 (77.3%)
Has overdosed personally	69 (71.1%)
# of overdoses [*median *(*IQR*)]	3 (2–7)

### Survey

Table 2 describes the main findings of willingness to use device solutions to detect and/or respond to a wearer’s overdose. In one question embedded in opioid use and overdose history, 76% of respondents indicated it is either likely (4) or very likely (5) that they would be willing to use a device with the functionality to detect if they have overdosed and deliver medication to reverse the overdose.

**Table 2:** Willingness to use various device interventions for opioid use harm reduction among study participants

Question	Likely (%)	Unlikely (%)	N total
Would you be willing to use a device that would help detect if you are suffering an opioid overdose and be able to give you a dose of a medication to treat the overdose	69 (76%)	22 (24%)	91
**While you are taking opioids, how likely would you be to wear each device?**			
A device that senses opioid overdose	70 (73%)	26 (27%)	96
A device that indicates the wearer is at risk of opioid overdose, like a medical ID	66 (69%)	29 (31%)	95
A device that straps naloxone to the body for a bystander to administer	52 (54%)	44 (46%)	96
A device that senses opioid overdose and administers naloxone, if needed	64 (67%)	32 (33%)	96
A device that alerts medical first responders that you have overdosed	68 (71%)	28 (29%)	96
A device that alerts bystander you may have overdosed	60 (63%)	35 (37%)	95
A device that monitors your vital signs	73 (77%)	22 (23%)	95
**For a wearable device to sense opioid overdose, how likely would you be to wear each device?**			
A necklace	48 (51%)	46 (49%)	94
A cannula (e.g., small tube under your nose)	12 (13%)	84 (88%)	96
Skin patch on chest	42 (44%)	54 (56%)	96
Skin patch on upper arm	53 (55%)	43 (45%)	96
Watch-appearing bracelet	72 (77%)	22 (23%)	94
Wrist bracelet	69 (73%)	26 (27%)	95
Shoulder strap	21 (41%)	30 (59%)	51
Thigh strap	15 (31%)	33 (69%)	48
Chest strap	22 (23%)	72 (77%)	94
Glasses	24 (26%)	70 (74%)	94
Knee brace	16 (33%)	32 (67%)	48
Ankle strap	44 (46%)	52 (54%)	96

In a section with a variety of proposed device-based solutions to limit the morbidity and mortality of overdose, a majority of survey participants responded that they were either likely or very likely to wear every proposed device. Reliance on bystander interventions was the least preferred of the device methods, with 54% responding they would likely wear a device that straps naloxone to their body for a bystander to administer, and 63% responding they would be likely to wear a device that senses opioid overdose and alerts a bystander. Solutions with purely monitoring functionality were the most preferred, with 77% responding likely to wear a device that monitors vital signs and 73% responding that they were likely to wear a device that senses opioid overdose. Reliance on trained medical intervention received intermediate responses, with 71% responding that they were likely to use a device that alerts medical first responders of an opioid overdose and 69% responding that they were likely to wear a device that indicates the wearer is at risk of overdose (like a medical ID). Finally, 67% of respondents indicated it is likely they would wear a device that senses an overdose and administers naloxone if needed. This likelihood remained consistent regardless of individual-level factors assessed based on Chi-squared analysis summarized in Additional file 2: Table 1.

Responses varied significantly between questions asking preferred location to wear a sensor. A watch-appearing bracelet (77%) and a wrist-like bracelet (73%) had the highest proportion likely to wear. A necklace (51%) and a skin patch on the upper arm (55%) were the only other locations that more than 50% of respondents indicated they were likely to wear an overdose-sensing device. Data regarding preferences for shoulder or thigh location for a device to automatically sense and reverse opioid overdose are separately presented in Additional file 3: Table 2.

### Interview

Themes that were identified in the open-ended responses to questions regarding the appearance of the device (“What factors are most important to the device’s appearance?”) are as follows:

*Discreetness* A large portion of participants highlighted the extent to which the device could be discreet as highly influential upon their likelihood of using it. This is related to two factors: (1) size of the device, as 89% of participants stated a preference for the smallest of the non-functional prototypes, and (2) concealment, with many participants describing blending with clothing and skin tone and having the appearance of a consumer “smart” device as preferred features.

*Comfort* Participants expressed the importance of the device being comfortable. One participant relayed the desire for a “soft, bendable” contact surface that would not cause discomfort during sleep if worn overnight. Others were discomforted by the texture of the box component and the straps of the non-functional prototypes.

*Indifference* A number of participants reported that if the device had the potential to reverse an otherwise fatal overdose, there is no aspect of the device’s appearance that would dissuade them from wearing it, citing the life-saving functionality of the proposed device.

Additional themes that were identified in the open-ended responses to questions regarding targeted distribution of the device (“Who needs this device the most? How should we get it to them?”) are as follows:

*Everyone* More than any specific subpopulation, participants expressed a need for a device to automatically sense and reverse opioid overdose for anyone with an ongoing opioid addiction. One participant said such devices should be “hand[ed] out like Narcan.” Another participant cited the danger of overdosing on a drug supply of unknown potency as a reason why the device would be widely useful: “You can overdose from 10 bags [of heroin] or 1 bag, you never know."

*People who are homeless* A number of participants highlighted people who are experiencing homelessness and use opioids as particularly vulnerable to overdose.

*People with low tolerance for opioids* Participants identified a number of populations at increased risk of overdose due to diminished tolerance and thus in greater need for the proposed device. These included people who had recently been released from jail or prison, people who had recently returned to use, and people coming to the Kensington-area from the surrounding tri-state area in order to use drugs, thus being unaccustomed to the potency of the local drug supply.

In open-ended conversations regarding the device concept, both overall positive and negative sentiments were present. Numerous participants were excited by the life-saving potential of the device and were confident such technology would keep them safer. Negative sentiments expressed by participants included aversion toward the possibility of “losing a high” after a false positive leads to reversal agent injection when overdose has not occurred, and inability to distinguish opioid overdose from non-opioid overdose leading to an ineffective reversal attempt. Some participants were also concerned of law enforcement misinterpretation of the device as a stash of drugs underneath clothing or a GPS monitoring device. Finally, some participants worried that a configuration of the device that alerted bystanders to an overdose would leave the person overdosing vulnerable to physical harm or robbery at the hands of ill-intentioned bystanders. A minority of participants believed that the device would be misplaced or stolen.

## Discussion

Our study revealed that people with a history of opioid use indicated broad support for a variety of devices with harm reduction capabilities. The broadest support was shown for devices with the least specific interventional functionality, such as those that monitor vital signs, sense opioid overdose, call medical authorities, and/or act as a medical ID, indicating the wearer is at risk of overdose. Additionally, participants maintained interest in a device that could itself sense an opioid overdose and administer naloxone to reverse the overdose. In contrast, devices relying on bystander intervention were the least supported solutions, despite the fact that the only currently available response to opioid overdose relies on others in the immediate vicinity. The results suggest that people with a history of opioid use show a strong desire and willingness to use device-based harm reduction solutions that limit morbidity and mortality of overdose as well as reduce the need for bystander intervention.

While willingness to use a device that could sense and reverse opioid overdose was strong, form factor was an important consideration. Configurations such as a cannula and glasses were strongly disfavored, while configurations such as a watch-appearing bracelet were highly favored. A minority of our sample population found a device like this likely to be stolen or lost, citing the device’s “street value”—that is, the perceived cost to end-users—as the most important factor that would determine whether it would be stolen. Similarly by free response, device obscurity was deemed an essential aspect to users; any device deemed too conspicuous was unacceptable. Concerns over privacy were further highlighted by many participants’ fear of suspicion from law enforcement regarding a device placed underneath clothing being mistaken for contraband. In addition, many participants also expressed comfort as another driving consideration. We believe addressing the identified user preferences will be crucial to the adoption of such a device among this population.

Collectively, the study supports the overall concept that people with OUD are willing to use a device that both senses and reverses opioid overdose. Such a device would address unmet needs for harm reduction by mitigating the long-term effects of opioid overdose and preventing fatal overdose, particularly among those who use alone [10–13]. While participants overwhelmingly identified a need for such a device among “anyone who uses opioids,'' certain subgroups were identified as having greater need, such as people who are experiencing homelessness or those with low tolerance after periods of abstinence (e.g., return to use after leaving rehabilitation centers, medication-assisted treatment, prison, or jail). However, this finding was inconsistent with a previous quantitative study that indicated that homelessness was negatively associated with willingness to wear a device to detect opioid overdose and alert bystanders [25]. We suspect the discrepancy may relate to our observation that some participants were distrustful of bystanders who may take advantage of their unconscious state during overdose, and thus preferred a closed-loop system. Populations experiencing homelessness and other high-need populations represent logical first beneficiaries of future distribution strategies. As fentanyl and other powerful synthetic opioids continue to spread through our communities, the need for a device that can quickly detect and respond to an opioid overdose will become even greater.

Efforts to distribute current overdose reversal options—including generic naloxone intramuscular injection, an auto-injectable intramuscular naloxone in the form of Evzio®, and intranasal naloxone in the form of Narcan®—have been remarkably successful [30, 31]. Reliable methods to detect opioid overdoses and expand bystander intervention as well as strategies to address the high proportion of fatal overdose among people who use alone remain an open question. Technical feasibility remains a significant concern, as the development of algorithmic approaches to reliably detect opioid overdoses, by monitoring blood oxygenation and motion activity for example, is a complicated task that must consider numerous possible sources of physiologic noise. Mechanisms for delivery of small-volume drug solutions, such as naloxone, through wearable devices must also be further explored. This study data can be used to guide solutions to the identified technical challenges, with particular attention to the importance of a discreet device.

There were limitations to our study. Our sample represents a population of people using opioids in an urban setting with one of the most extensive naloxone distribution efforts in the USA, with hundreds of thousands of Narcan kits distributed in the past few years [32]. This may have influenced the perception our study sample had of the need for the devices we proposed and limit generalizability of our findings. Additionally, among those whose housing status was known, the vast majority of our respondents were experiencing housing insecurity, while the majority of fatal overdoses in Philadelphia occur among individuals in their own homes [26]. The effects of housing status, such as those observed by Ahamed et al. (2019), could not be assessed due dynamic living conditions among those surveyed due to changing COVID-19 policies on shelter capacity [25]. Furthermore, as the data were collected during the COVID-19 pandemic while overdoses rose rapidly among changing demographic groups, the potential effects of the pandemic on attitudes toward new overdose reversal interventions and propensity for high-risk drug use behaviors were not captured in the scope of our questions [23]. Recent stressors, such as the need for social distancing considerations and mental wellness strain due to the COVID-19 pandemic, may increase the rate of solitary opioid use, as well as increase the rates of return to use from settings providing medication for opioid use disorder (MOUD) [5, 26, 33]. Future directions include surveys of populations that more broadly represents those at highest risk of overdose as well as deeper exploration of form factor expectations.

## Conclusions

People with a history of opioid use show a desire and willingness to use device-based harm reduction solutions but are skeptical of bystander involvement. Support for a device with the capabilities to sense and reverse an opioid overdose was robust, although form factor was noted as an essential consideration for safety from both law enforcement and bystander harm. With multiple device-based solutions for opioid use underway, innovators must keep in mind the preferences of people using opioids themselves.

### Supplementary Information

**Additional file 1.** **Figure 1.** Non-functional looks-like prototype design.

**Additional file 2.** **Table 1.** Individual-levelfactors affecting willingness to use a wearable device to sense and reverse opioid overdose.

**Additional file 3.** **Table 2.** Usability factors for a wearable device to sense and reverse opioid overdose.

## Data Availability

The datasets collected and analyzed during the current study are available from the corresponding author on reasonable request.

## Appendix 1

Survey. Questions 30, 31, 38, 39, and 42 were not answered by all participants because they were added part way through the execution of the study.

## Appendix 2

Semi-structured interview questions.

## Abbreviations

CDC: Centers for Disease Control and Prevention
OUD: Opioid use disorder
PPP: Prevention Point Philadelphia
