# Supplementary material for: Willingness to use a wearable device capable of detecting and reversing overdose among people who use opioids in Philadelphia
Source: Harm Reduct J. 2021 Jul 23;18:75. doi: 10.1186/s12954-021-00522-3 (PMC8299455; doi:10.1186/s12954-021-00522-3)
Supplement: Supplementary file 1 — Additional file 1. Figure 1. Non-functional looks-like prototype design. [file 12954_2021_522_MOESM1_ESM.docx]

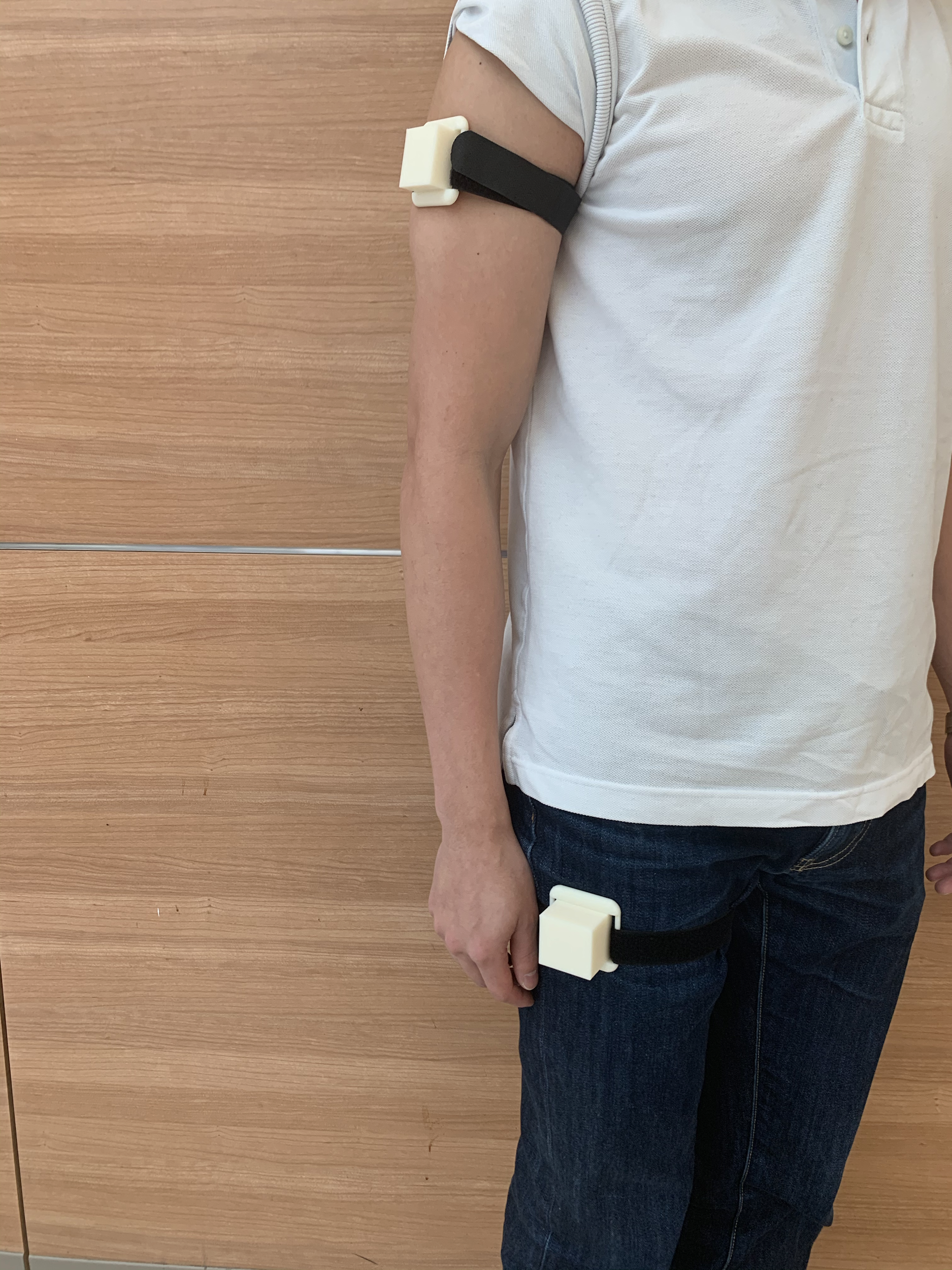

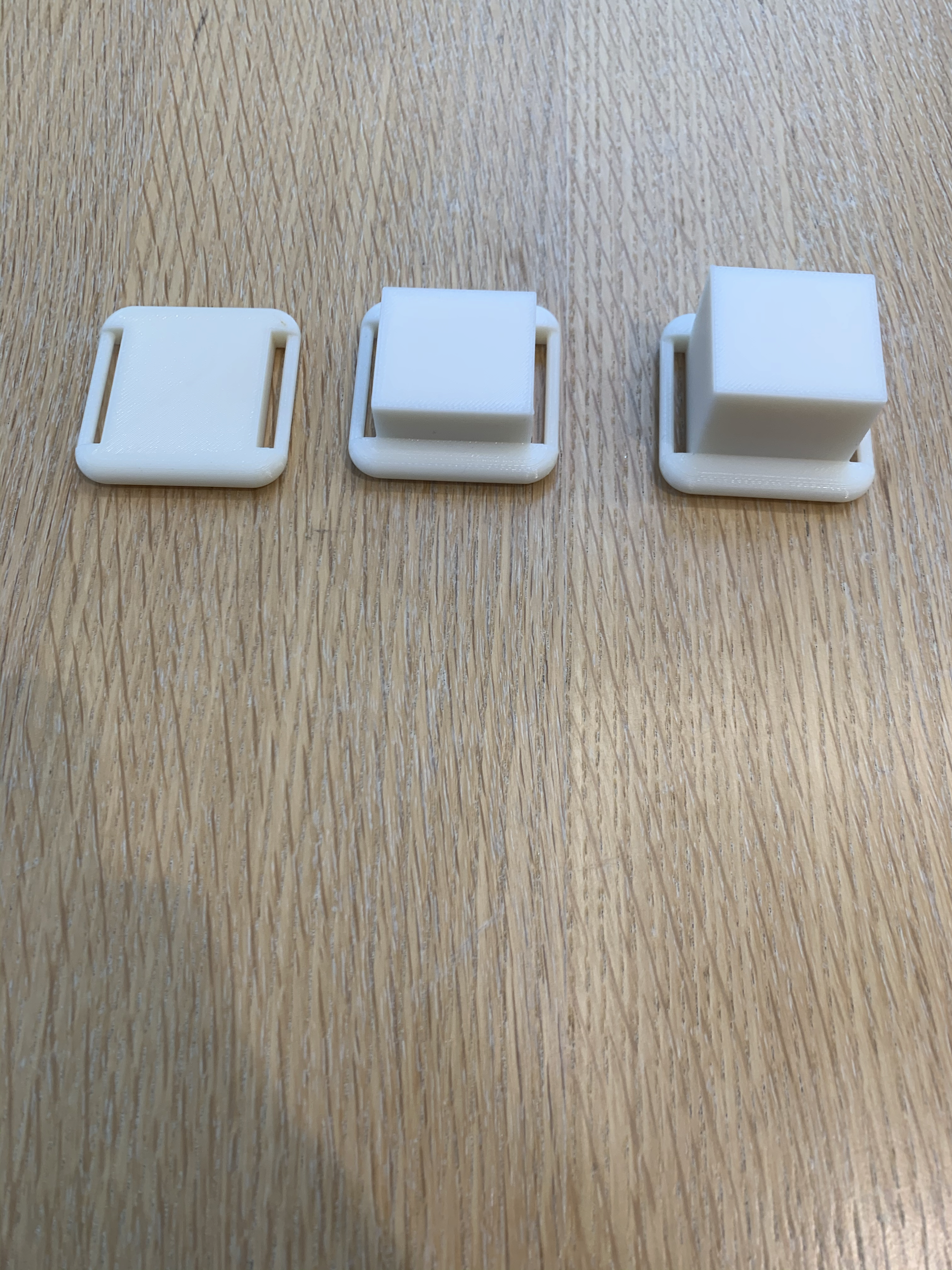


**Supplementary Figure 1.** Non-functional prototypes presented during the semi-structured interview. Top: The prototypes were presented to each participant and strapped to shoulder and thigh sites to mimic the tactile experience of wearing an automated overdose reversal device. Bottom: Each prototype was a 1”x1”x1/8” volume with an additional 0.5” height for the medium-sized prototype and an additional 1” height for the large-sized prototype.
